# Cognitive disorders in diabetes

**DOI:** 10.3389/fcdhc.2025.1534105

**Published:** 2025-02-24

**Authors:** Karim Gariani, Dongryeol Ryu, Manfredi Rizzo

**Affiliations:** ^1^ Service of Endocrinology, Diabetes, Nutrition, and Therapeutic Education, Faculty of Medicine, Geneva University Hospitals, Geneva, Switzerland; ^2^ Diabetes Center of the Faculty of Medicine, University of Geneva Medical School, Geneva, Switzerland; ^3^ Department of Biomedical Science and Engineering, Gwangju Institute of Science and Technology, Gwangju, Republic of Korea; ^4^ Promise Department, School of Medicine, University of Palermo, Palermo, Italy; ^5^ College of Medicine, Ras Al Khaimah Medical and Health Sciences University, Ras Al Khaimah, United Arab Emirates

**Keywords:** diabetes, cognitive impairment, dementia, public health, diabetes-related complication, elderly

## Introduction

Diabetes is increasingly recognized as a condition with far-reaching implications, including its impact on cognitive health (1). Early observations of the relationship between diabetes and cognitive impairment date back over a century, initially based on simple neuropsychological assessments comparing individuals with and without diabetes. Since then, large-scale epidemiological studies, advanced imaging technologies, and neuropathological investigations have solidified this association, revealing a complex interplay between metabolic dysregulation and cognitive decline (2, 3).

Today, it is well-established that individuals with type 2 diabetes face a 1.6-fold greater risk of developing dementia compared to their non-diabetic peers. Estimates suggest that cognitive impairment affects between 20% and 70% of people with diabetes, depending on the study and population examined (4). Beyond its impact on daily living, cognitive decline in diabetes is associated with a higher risk of cardiovascular events and mortality Today, it is well-established that individuals with type 2 diabetes face a 1.6-fold greater risk of developing dementia compared to their non-diabetic peers. Estimates suggest that cognitive impairment affects between 20% and 70% of people with diabetes, depending on the study and population examined (4). Beyond its impact on daily living, cognitive decline in diabetes is associated with a higher risk of cardiovascular events and mortality (5). The relationship between diabetes and cognitive disorders is shaped by multiple interacting factors, among which socioeconomic status plays a crucial role. Education level, a key determinant of socioeconomic status, has been widely studied and shown to influence the risk of developing diabetes. Individuals with higher education tend to have a lower risk, while those with lower education are more susceptible to lifestyle-related diseases such as hypertension, obesity, and diabetes. Since these conditions are also associated with cognitive decline, individuals from lower socioeconomic backgrounds may experience a more severe and rapid deterioration of cognitive functions when living with diabetes (6). This interconnected relationship suggests that socioeconomic disparities not only increase the risk of diabetes but also contribute to an accelerated cognitive decline through the prevalence of other lifestyle-related diseases.

Of note, the recent COVID-19 pandemic has demonstrated that infected patients tend to develop higher levels of anxiety and depression, as well as an increased risk of cognitive decline. Given that individuals with diabetes are at greater risk of severe COVID-19 infections, it can be hypothesized that this infection may also directly or indirectly worsen and amplify cognitive impairment in diabetic patients (7). However, the precise impact of COVID-19 on cognitive disorders in individuals with diabetes remains to be fully established.

For elderly individuals, particularly those with coexisting dementia, managing diabetes effectively becomes increasingly challenging. Given the growing global prevalence of diabetes and projections pointing to an even steeper rise in the coming decades, diabetes-related cognitive disorders are emerging as a critical public health challenge. These conditions not only diminish quality of life for millions worldwide but also impose substantial economic burdens on healthcare systems through both direct costs, such as treatments, and indirect costs, like lost productivity and caregiving expenses.

## Current situation: a vicious cycle

Cognitive impairment is now acknowledged as a significant complication of diabetes. It encompasses a spectrum of disorders, from mild cognitive decline to severe dementia, which profoundly affect individuals’ memory, attention, and decision-making abilities. These impairments complicate everyday activities, particularly the self-management of diabetes, creating a feedback loop of worsening cognitive and glycemic control (8). Patients with diabetes and cognitive dysfunction often experience behavioral and psychological challenges that further impede their ability to follow medical advice, adhere to treatment regimens, and maintain consistent blood glucose monitoring. These difficulties lead to reduced treatment adherence, less frequent monitoring, and greater glycemic variability. The cascading effects include recurrent episodes of hypo- and hyperglycemia, which exacerbate both cognitive and metabolic dysfunction These impairments complicate everyday activities, particularly the self-management of diabetes, creating a feedback loop of worsening cognitive and glycemic control (8). Patients with diabetes and cognitive dysfunction often experience behavioral and psychological challenges that further impede their ability to follow medical advice, adhere to treatment regimens, and maintain consistent blood glucose monitoring. These difficulties lead to reduced treatment adherence, less frequent monitoring, and greater glycemic variability. The cascading effects include recurrent episodes of hypo- and hyperglycemia, which exacerbate both cognitive and metabolic dysfunction (9). This vicious cycle is compounded by the inherently demanding nature of diabetes management. Decision-making about insulin dosing, meal planning, physical activity, and medication adherence is complex, requiring consistent cognitive effort throughout the day. For patients with impaired cognitive function, these daily demands often prove overwhelming, increasing their risk of complications and diminishing their quality of life (10).

Research has uncovered several interconnected factors contributing to the dual burden of diabetes and cognitive impairment. These include poor glycemic control, frequent episodes of hypoglycemia, advanced age, coexisting conditions such as depression, and the detrimental effects of social isolation. Each of these factors not only exacerbates the challenges faced by patients but also complicates their management. Despite the significant impact of these issues, awareness and understanding among healthcare professionals remain inadequate. Many patients report feeling unsupported and misunderstood when attempting to address their cognitive challenges, emphasizing the urgent need for enhanced education and specialized training for healthcare providers (3).

Adding to the complexity, diabetes-related cognitive impairment often develops insidiously, with early and subtle signs manifesting during middle age or, in some cases, even before the age of 40 (9). These early symptoms are frequently overlooked or attributed to other causes, delaying both diagnosis and intervention. This highlights the critical importance of implementing proactive screening and early detection strategies, which could significantly mitigate the progression of cognitive decline and improve patient outcomes.

However, despite growing recognition of this issue, the integration of cognitive disorders into diabetes management guidelines is a relatively recent advancement. While this represents a positive step forward, its application in routine clinical practice remains inconsistent and fragmented. To bridge this gap, greater efforts are needed to standardize approaches, incorporate cognitive assessments into diabetes care, and ensure that healthcare teams are equipped with the tools and knowledge necessary to address this complex interplay effectively. This will require not only improved awareness but also a commitment to reshaping care models to better serve patients living with this dual burden.

## Addressing uncertainties and charting the future

Despite growing awareness, significant gaps remain in the management of cognitive impairment in individuals with diabetes. Current clinical practices lack clear consensus on key issues, including which screening tools are most effective, how frequently cognitive assessments should be performed, and what thresholds should prompt further intervention. While the American Diabetes Association (ADA) recommends annual cognitive screening for individuals aged 65 and older, no specific guidance exists for younger adults, despite evidence that cognitive decline can occur in this population as well (11).While the American Diabetes Association (ADA) recommends annual cognitive screening for individuals aged 65 and older, no specific guidance exists for younger adults, despite evidence that cognitive decline can occur in this population as well (11).

Emerging therapies offer potential hope. Recently, the FDA approved two anti-amyloid monoclonal antibodies for the treatment of early Alzheimer’s disease. While these treatments are considered a promising development, their role in elderly diabetic populations remains unclear. Similarly, GLP-1 receptor agonists (GLP-1RAs), widely used in diabetes management, have shown promising neuroprotective effects in preclinical studies of neurodegenerative diseases (12, 13). However, their precise role in preventing or mitigating cognitive decline in diabetic patients has yet to be determined. In addition, increasing evidence suggests that the use of sodium-glucose cotransporters-2 inhibitors, another class of novel anti-diabetic therapeutic agents, may be associated with improved cognitive deficits, although the exact mechanisms involved are still not fully elucicated (14).

Most existing guidelines are based on expert opinion rather than robust clinical data, underscoring the need for more comprehensive research. Longitudinal studies with larger cohorts and extended follow-up periods are essential to establish evidence-based practices.

Technological advances, particularly in continuous glucose monitoring (CGM), offer promising solutions for improving glycemic control and mitigating the risk of cognitive decline. CGMs enable real-time tracking of blood glucose levels, providing critical insights into glycemic variability (15, 16). However, their accuracy remains limited during extreme hypoglycemia, and more research is needed to optimize their use in patients with cognitive impairments.

## A call to action

To effectively address the dual burden of diabetes and cognitive impairment, a collaborative, multidisciplinary approach is not just beneficial, it is essential. This approach should involve a diverse team of healthcare professionals, including endocrinologists, dementia specialists, primary care physicians, diabetes educators, dietitians, and physical therapists. By pooling their expertise, such a team can create a comprehensive care model that optimizes the use of resources, customizes treatments to meet the unique needs of each individual, and ensures the seamless integration of both pharmacological and non-pharmacological strategies.

In addition to the direct care provided by healthcare teams, professional societies hold a pivotal role in catalyzing systemic change. These organizations can spearhead broad-based educational initiatives aimed at healthcare providers, focusing on raising awareness about the intersection of diabetes and cognitive disorders. Such efforts can include the development of targeted, patient-centered educational materials that simplify complex information, making it accessible and actionable for individuals and families affected by these conditions.

Moreover, fostering stronger collaboration among various specialties and care disciplines is critical to addressing the multifaceted challenges posed by this dual burden. Increased investment in these initiatives, both in terms of funding and strategic planning, can significantly enhance the ability of both patients and healthcare providers to manage the overlapping impacts of diabetes and cognitive decline. Through such concerted efforts, the healthcare system can move toward a more proactive and holistic approach, ultimately improving outcomes and quality of life for those affected.

## Conclusion

The link between diabetes and cognitive impairment represents a growing public health crisis. The interplay between glycemic control and cognitive decline creates a self-perpetuating cycle that undermines patients’ ability to manage their condition effectively. Addressing this issue requires a concerted effort to enhance awareness, deepen our understanding of the underlying mechanisms, and develop tailored therapeutic strategies. New technologies are already playing a crucial role in diabetes management and could offer significant support in the care of older adults with cognitive decline. They enable a more efficient response to fluctuations in the patient’s blood glucose levels and medication needs, provide caregivers with remote monitoring capabilities for faster intervention during patient crises, and assist in adjusting and updating long-term treatment plans based on patterns observed in the data collected. Furthermore, these technologies can help evaluate treatment adherence and self-management.

As the global population continues to age and obesity rates rise, the prevalence of diabetes-related cognitive disorders is set to increase dramatically. This growing burden calls for immediate action from healthcare providers, researchers, and policymakers alike. By investing in multidisciplinary care, advancing research, and promoting awareness, we can mitigate the impact of this dual challenge and improve outcomes for millions of individuals worldwide.
